# Conservative Treatment for Right Ventricular Free Wall Rupture in a Patient with Acute Myocardial Infarction

**DOI:** 10.1155/2020/8836627

**Published:** 2020-07-24

**Authors:** Mohd Al-Baqlish Mohd Firdaus, Nur Syahirah Abdul Rahim, Nurtasneem Rusdi, Nor Saadah Idris, Mohd Ridzuan Mohd Said, Imran Zainal Abidin

**Affiliations:** ^1^Division of Cardiology, Department of Medicine, University Malaya Medical Centre, 59100 Kuala Lumpur, Malaysia; ^2^Department of Internal Medicine, Kulliyyah of Medicine, International Islamic University Malaysia, 25200 Kuantan, Pahang, Malaysia; ^3^Department of Radiology, University Malaya Medical Centre, 59100 Kuala Lumpur, Malaysia

## Abstract

Ventricular wall rupture possesses a high mortality rate in patients with acute myocardial infarction. We presented a case of a ninety-year-old gentleman who presented with acute inferolateral myocardial infarction in cardiogenic shock and right ventricular free wall rupture. He was treated conservatively and survived.

## 1. Introduction

Cardiac rupture is a major complication following acute myocardial infarction (AMI) apart from ventricular fibrillation and cardiogenic shock [1]. Although the incidence was reduced with the practice of reperfusion therapy, yet it still carries a high mortality rate of more than 50% [2]. Cardiac rupture may involve free wall of ventricles, the interventricular septum, and atrium or papillary muscles, in which cases of free wall rupture (FWR) are approximately ten times less frequent compared to the septal and papillary muscle rupture [2–4]. Left ventricular rupture accounts for the majority of ventricular rupture cases reported, while isolated right ventricular free wall rupture (RVFWR) is a rare entity with very few cases reported previously [3]. Here, we presented a case of RVFWR in cardiogenic shock secondary to AMI, treated conservatively and survived.

## 2. Case Report

**Figure 1:**
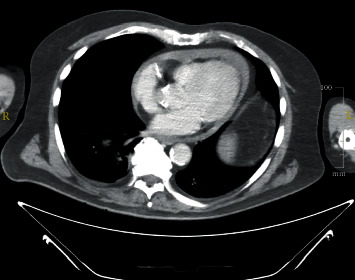
CT scan showed the presence of global pericardial effusion.

**Figure 2:**
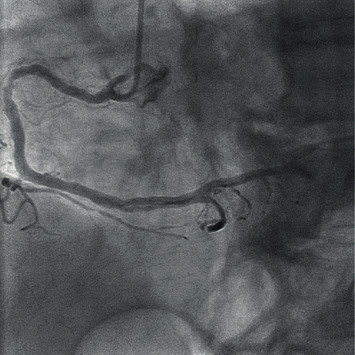
Presence of ulcerated plaque at the proximal right coronary artery with total occlusion of the posterior descending artery and posterolateral branch artery.

A ninety-year-old gentleman with underlying dementia and hypertension was presented with a sudden onset of central chest pain while watching television. Upon arrival at the emergency department, he was in severe pain and sweaty. Initial blood pressure was 90/50 mmHg with a heart rate of 110 beats per minute. ECG showed acute inferolateral myocardial infarction. On cardiovascular examination, there was no clinical sign of cardiac tamponade, and the auscultation of the lung was clear. Bedsides, echocardiography was performed showing pericardial effusion with a maximum diameter of 1.3 cm over the apex with no features of cardiac tamponade. The inferior lateral wall was hypokinesia, and the right ventricle wall was akinetic. Given the ECG and echocardiographic findings, and the patient was in severe pain, we decided to proceed with CT aortogram to rule out aortic dissection. The CT scan showed no evidence of aortic dissection; however, there was a presence of hemopericardium (Figure 1). We decided to proceed with a primary percutaneous coronary angiogram (Figure 2).

**Figure 3:**
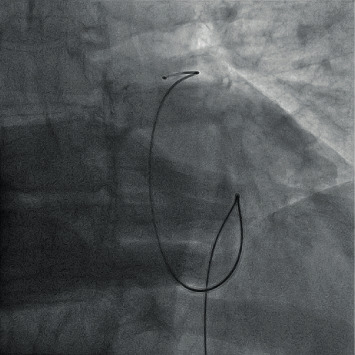
A guidewire was being inserted into the pericardium.

The patient was persistently hypotensive and did not respond to fluid resuscitation and inotropic support. We decided to abundant the angioplasty procedure and proceeded with emergency pericardial tapping (Figure 3). 350 ml of blood was drained from the pericardium. The hypotension resolved after the procedure, and we were able to off the inotropic infusion. The pericardial drainage was in situ for three days and drained haemoserous fluid. After removal of the pericardial drainage, there was a reaccumulation of pericardial effusion. We referred the patient to the cardiothoracic surgeon, but the patient and family opted for conservative treatment and refused for any invasive or surgical intervention. The patient was not given heparin throughout the hospital stay and discharged home with a single antiplatelet. The proximal right coronary artery was stented three months later. He was last seen in our clinic in December 2019 and is currently doing well. The echocardiogram was repeated, and it illustrates mild left ventricular dysfunction with an ejection fraction of 47%. There was no significant residual pericardial effusion. The right ventricular function was normal.

## 3. Discussion and Literature Review

Ventricular rupture is a rare but fatal mechanical complication of AMI. Most cases were associated with death following cardiac tamponade and cardiogenic shock [5, 6]. The incidence of ventricular rupture secondary to AMI was reported between 2 and 4% and responsible for 10-15% of hospital death [2, 4, 6]. Data from the SHOCK registry showed that the incidence of ventricular wall rupture presented as a cardiogenic shock was 3.9% and the mortality rate was 87.3% [7]. About 80-90% of cases involved the left ventricle, while cases of isolated postinfarction RVFWR appeared to be extremely rare since most literature was referring the cases as an extension of ventricular septal rupture [2, 3, 8]. Isolated RVFWR is mainly caused by inferolateral MI and right coronary artery occlusion [9, 10]. It was postulated that very low incidence of RVFWR was due to lower pressure effect over the right ventricle and it rarely undergoes transmural infarction [3, 6].

Previous literatures demonstrated that patients with advanced age, female, and concomitant hypertension had a higher risk of cardiac rupture [2, 3, 5, 6]. Some authors found that patients with the first episode of AMI, especially involving the massive infarction of anterior or lateral wall with high ST elevation, tend to develop this complication [2, 3, 9]. On the other side, any delay in the administration of treatment after the onset of the symptom was identified as one of the major risk factors of cardiac rupture along with the thinned ventricle wall, less collateral circulation, and disfiguration of elastic tissue after transmural MI [9, 10].

Early detection and management were crucial to reduce patients' mortality. Physicians need to be aware regarding the signs and symptoms of ventricular rupture or impending rupture in patients with AMI. Generally, it includes loss of consciousness, facial cyanosis, bradycardia, and hypotension following pericardial haemorrhage. Such patients may also complain of chest pain which is resistant even to opiates, agitation, and recurrent emesis. These may also be associated with muffled heart sound, pericardial rub, pulsus paradoxus, cardiac tamponade, shock, or asystole [2, 5]. A review done by Bajaj et al. [11] showed that 70% of cases of acute ventricular free wall rupture presented with sudden cardiac death. In contrast, in a few cases, it can be a gradual or incomplete rupture with slow or recurrent bleeding into the pericardium, causing progressive or recurrent cardiac tamponade, which is termed as subacute ventricular free wall rupture. In such cases, they usually presented with hypotension with or without cardiogenic shock, and urgent intervention may be life-saving [2, 11]. Apart from that, a higher survival chance may be expected if a small rupture limits the haemorrhage, a tortuous tract of dissection within the ventricular muscle, or the formation of a seal by fibrin clots or pericardial tissue [5]. Referring to our case, it is an acute isolated RVFWR, and it can be speculated that a small-sized rupture and formation of thrombus limit the amount of blood extravasated into the pericardium, hence making the free wall rupture without surgical repair compatible with long-term survival [10].

The findings of electromechanical dissociation and echocardiographic or radiological evidence of pericardial haemorrhage favour the diagnosis of ventricular free wall rupture [12]. In clinically suspected rupture, transthoracic echocardiography is a fast and sensitive test to confirm the diagnosis. However, there might be some limitations in evaluating the right ventricle because of its crescent shape, substernal location, and the presence of a large amount of artefact [6, 9]. Fortunately, in our case, the echocardiographic finding of pericardial effusion and the evidence of right coronary artery occlusion and its branches on primary percutaneous coronary angiogram brought us to the diagnosis of RVFWR after excluding aortic dissection by CT aortogram. Previously cited by Bajaj et al. [11], the presence of echocardiographic finding of pericardial effusion greater than 5 mm with intrapericardial echoes in a hypotensive patient carries 90.9% sensitivity for cardiac rupture, and it is associated with 43% of 30-day mortality.

In managing ventricular rupture, available treatment options include conservative measures and salvageable surgery. However, currently, there are no specific guidelines available in determining patient criteria and specific timing for surgical intervention [13]. Conservatively, patients were treated to achieve hemodynamic stability, and this includes fluid management, inotrope support, and reperfusion therapy. As cited by Dandeniyaarachci et al. [3] and Bajaj et al. [11], the incidence of cardiac rupture can be reduced with reperfusion therapy; however, the risk increases if thrombolysis is being done after 14 hours of symptom onset. The rationale behind treating patients is conservatively mainly due to the tamponade effect created by the thrombus that previously formed from extravasating blood in which only applicable for a small-sized rupture [10].

Some authors reported that surgery is the only salvageable procedure and superior to conservative measures once hemopericardium is confirmed [1, 2]. Most of the salvaged cases reported have been treated only by closure of the rupture site [14]. Few techniques have been described, including direct compression, suturing with pledgets, and sutureless patch glue [3]. With early surgical treatment, it reduced mortality with the survival rate of 85% after a successful repair [3, 7].

With regard to our case, an initial referral was made for surgical intervention. However, upon the patient's request, he was treated conservatively, and surprisingly, he survived and well. A similar case was being reported by Sherer et al. [10], and this remote phenomenal can be explained by quick resolution of the thrombus along with the small myocardial defect that later became impermeable to the blood. However, the recurrent rate of right ventricular rupture was not well established previously; therefore, regular follow-up with the timely echocardiogram is essential in detecting any recurrence or presence of complications.

## 4. Conclusion

Postinfarction RVFWR is a rare entity with a fatal outcome. Therefore, early detection and prompt interventions are life-saving and crucial in reducing the mortality rate.

## Data Availability

The patient clinical case note is available at the medical record office, University Malaya Medical Centre.
